# Intravenous bisphosphonate therapy and atrial fibrillation/flutter risk in cancer patients: a nationwide cohort study

**DOI:** 10.1038/bjc.2011.338

**Published:** 2011-08-30

**Authors:** R Erichsen, C F Christiansen, T Frøslev, J Jacobsen, H T Sørensen

**Affiliations:** 1 Department of Clinical Epidemiology, Aarhus University Hospital, Olof Palmes Allé 43-45, 8200 Aarhus N, Denmark

**Keywords:** atrial fibrillation, diphosphonates, neoplasms, risk

## Abstract

**Background:**

There is conflicting evidence regarding bisphosphonates and atrial fibrillation (AF) risk in osteoporosis patients. However, bisphosphonates are used in much higher doses in treatment of bone metastasis and hypercalcemia, but little is known about the AF risk in cancer patients.

**Methods:**

We conducted a nationwide population-based cohort study using Danish databases. All cancer patients exposed to intravenous bisphosphonates during 2000–2008 were matched with two non-exposed cancer patients by cancer type, distant metastasis presence at diagnosis, age, and gender. We used Cox proportional hazard regression to estimate hazards ratios (HRs) of AF/flutter adjusting for important confounding factors.

**Results:**

Of the 3981 cancer patients exposed to intravenous bisphosponates, 128 (3.2%) developed AF/flutter. This condition occurred in 192 (2.4%) of the 7906 non-exposed cancer patients, corresponding to an adjusted HR of 1.7 (95% CI: 1.2–2.4).

**Conclusion:**

Intravenous bisphosphonates may increase AF/flutter risk in cancer patients.

Bisphosphonates inhibit osteoclast-mediated bone resorption and are widely prescribed for prevention and treatment of osteoporosis (Woolf and Akesson, 2003). Bisphosphonates are now also used in treating bone metastasis and hypercalcemia in cancer patients (Coleman *et al*, 2008). Unexpectedly, a clinical trial (Black *et al*, 2007) followed by two observational studies (Heckbert *et al*, 2008; Abrahamsen *et al*, 2009) and two meta-analyses (Loke *et al*, 2009; Bhuriya *et al*, 2010) found that bisphosphonates may be associated with increased risk of atrial fibrillation (AF). In contrast, results of several other clinical trials, observational studies, and meta-analyses have been less supportive of increased AF risk in patients treated with bisphosphonates (Karam *et al*, 2007; Lyles *et al*, 2007; Sorensen *et al*, 2008; Bunch *et al*, 2009; Grosso *et al*, 2009; Mak *et al*, 2009; Huang *et al*, 2010; Kim *et al*, 2010; Vestergaard *et al*, 2010). In addition, little is known about the potential underlying mechanism. Most studies were based on osteoporotic patients, with few examining the association in cancer patients (Wilkinson *et al*, 2010; Arslan *et al*, 2011). In cancer patients, bisphosphonates are administered in doses up to 10 times higher than those used for osteoporosis treatment. Thus cancer patients may be at particularly increased risk of AF, as indicated by one study (Wilkinson *et al*, 2010). In addition, cancer patients are often exposed to chemotherapies that itself increase this risk. The limited and conflicting available evidence led us to conduct a large nationwide follow-up study of AF and flutter risk in Danish cancer patients treated with bisphosphonates.

## Materials and methods

We used the Danish Cancer Registry (DCR) to identify all patients with incident cancers between 1 January 2000 and 31 December 2008. The DCR hold records on all incident malignant neoplasms in Denmark since 1943, including patients’ civil registration number, month and year of cancer diagnosis, cancer type, and tumour spread at diagnosis (Storm *et al*, 1997). The unique civil registration number assigned to all Danes at birth or upon immigration (Pedersen *et al*, 2006) allowed us to link each cancer patient to the Danish National Registry of Patients (DNRP) to obtain information on intravenous (IV) bisphosphonate therapy around the time of cancer diagnosis (excluding patients receiving bisphosphonate therapy more than 30 days before their cancer diagnosis) and on medical history. The DNRP include all non-psychiatric hospitalisations in Denmark since 1977 and hospital outpatient contacts since 1995. Its records include dates of admission and discharge, treatment and procedure codes (BWHB40 for bisphosphonates), and up to 20 diagnoses coded by physicians according to the *International Classification of Diseases* (ICD), 10th revision since 1994 (Andersen *et al*, 1999).

We matched each bisphosphonate-exposed cancer patient to two non-exposed cancer patients, by cancer type, presence of distant metastasis at diagnosis, age, and gender. We defined the index date as the date of the first bisphosphonate treatment code for bisphosphonate-exposed patients and the corresponding date in the matched non-exposed cancer patients. We followed the cancer patients from their index date until an AF/flutter hospital discharge diagnosis, death, emigration, or the end of study (25 January 2010), whichever came first. Patients with AF/flutter were identified from the DNRP using ICD-10 code I48.9. Although this code includes both AF and flutter, only 5% of patients have isolated atrial flutter (Frost and Vestergaard, 2004). Patients diagnosed with AF/flutter before their index date were excluded. We used stratified Cox proportional hazard regression analysis to estimate hazard ratios (HRs) as an estimate of the relative risk of AF/flutter in bisphosphonate-exposed compared with non-exposed cancer patients, adjusting for the following potential confounding factors extracted from the DNRP: cardiovascular disease, diabetes, pulmonary disease, hyperthyroidism, alcoholism, and renal failure (refer Christiansen *et al*, 2009 for ICD-10 codes) and the receipt of surgery (surgery code K) and chemo- and radiotherapy (treatment codes BWG and BWHA) in the year before the index date.

In sub-analyses we restricted to cancer patients living in Central and Northern Denmark (population of 1.8 millions) with prescription data stored in the Aarhus University Prescription Database (Ehrenstein *et al*, 2010). This database includes the type and amount of the drug prescribed according to the Anatomical Therapeutic Chemical classification system, the date the drug was dispensed, and the patient's civil registration number for reimbursed prescriptions. We used the same methodology as above, further adjusting for medication use as a marker of underlying disease associated with AF/flutter prescribed in the year before index date (cardiovascular, respiratory, or antithyroid drugs, thyroid hormone replacement therapy, non-steroid inflammatory drugs or COX-2 inhibitors, vitamin K antagonists, and oral glucocorticoid drugs (Christiansen *et al*, 2009 for codes)). We also did an analysis excluding digoxin users, which are likely to represent prevalent AF/flutter, and another analysis excluding oral bisphosphonate users.

## Results

We identified 3981 cancer patients exposed to IV bisphosphonate therapy and 7906 non-exposed cancer patients and followed these patients for a total of 22 642 years. In both exposed and non-exposed patients, the median age was 65 years, 61% were women, and 23% had distant metastasis at cancer diagnosis (Table 1). The most frequent cancer types were breast cancer and multiple myeloma and potential confounding factors were almost equally distributed between the bisphosphonate-exposed *vs* non-exposed groups except for surgery, chemo-, and radiation therapy (Table 1). Among cancer patients exposed to IV bisphosphonate therapy, 128 (3.2%) developed AF/flutter after a median time from index of 335 days and a medium time from latest treatment of 63 days. Among non-exposed patients, 192 (2.4%) developed this condition. This corresponds to a crude HR of 1.8 (95% CI: 1.4–2.4) and an adjusted HR of 1.7 (95% CI: 1.2–2.4).

In the analysis restricted to Central and Northern Denmark and further adjusting for medication use the crude HR was 1.5 (95% CI: 0.9–2.4) and the adjusted HR was 1.4 (95% CI: 0.9–2.3). Exclusion of digoxin and oral bisphosphonate users did not change the estimates (results not shown).

## Discussion

Our study showed that cancer patients exposed to IV bisphosphonates are at increased risk of AF/flutter. This is consistent with a recently published study using the Surveillance, Epidemiology and End Results (SEER) Medicare database (Wilkinson *et al*, 2010). In the SEER study, a total of 6857 bisphosphonate users were matched according to cancer type, age, sex, presence of bone metastasis, and SEER region to 13 714 bisphosphonate non-users in the 1995–2003 period (age ⩾65 years). The study reported an HR of 1.30 (95% CI: 1.18–1.43) for AF among bisphosphonate users compared with non-users, together with an increased risk of stroke. In contrast, a Turkish cross-sectional study, from a single outpatient clinic, of 124 patients with solid tumours and bone metastasis treated with IV bisphosphonates did not detect any AF/flutter episodes (Arslan *et al*, 2011). However, that study was clearly underpowered and its design precluded evaluation of new onset of AF/flutter. In all other studies, examination of the association between bisphosphonates and AF was restricted to osteoporosis patients. These studies, comprehensively outlined in the 2010 review by Howard *et al* (2010), had inconsistent findings for AF risk. A 2010 communication issued by the US Food and Drug Administration, based on the results from some of these studies and subsequent analyses, also did not identify a clear association (FDA, 2010). Nevertheless, even a small association between bisphosphonates and AF (perhaps insignificant in osteoporosis patients) may be important for cancer patients as this vulnerable group receives doses of bisphosphonates up to 10 times higher than those given to osteoporosis patients.

The strengths of our study included being population based and conducted in a nationwide setting within a free tax-supported healthcare system, with access to complete and valid data on cancer (Storm *et al*, 1997). These features reduced the risk of selection, referral, and information biases and its results are generalisable to patients of all ages with cancer at any site. Furthermore, even though we did not identify AF/flutter directly by ECG but by discharge codes in the DNRP, the coding had a positive predictive value estimated to be as high as 97% (Frost and Vestergaard, 2004). Still, our study has limitations. Although coding of IV bisphosphonate use in the DNRP has high predictive value, it may be incomplete. This could cause exposed cancer patients to be misclassified as non-exposed, leading to underestimation of the relative risk. In addition, the specific subtype or dose of bisphosphonates was not recorded in the DNRP, precluding investigation of any dose-response or risk differences between the bisphosphonate types registered for IV administration in Denmark (zoledronic acid, ibandronic acid, and pamidronate). Although Danish guidelines recommend referral of all patients with incident AF/flutter to a hospital clinic, we may have excluded some prevalent cases only seen by a general practitioner. Nevertheless, this is unlikely to bias our results, because AF/flutter diagnoses are probably not missed more often in exposed cancer patients than in unexposed patients. Furthermore, we may have missed some AF/flutter transient cases as they probably would be less likely to be recorded in the DNRP and we were also unable to report on the severity of AF/flutter.

Confounding remains a potential source of bias in observational studies. The matching in our study resulted in almost equal distribution of several important confounding factors between exposed and non-exposed cancer patients. Despite this, there may still be residual confounding (e.g. from smoking) and patients at an *a priori* increased risk of AF/flutter might have been more or less likely to be treated with bisphosphonates (confounding by indication). For instance, most cancer patients exposed to bisphosphonates have bone metastases and thus might represent more severely ill patients with a higher risk of AF than unexposed patients, causing us to overestimate the HR.

Both clinical trials and observational studies are susceptible to bias, and neither provides perfect information (Sorensen *et al*, 2006), as indicated by previous studies on the association between bisphosphonates and AF. However, until further data become available, our data suggest that clinicians should be aware of the increased risk of AF/flutter in cancer patients treated with IV bisphosphonates.

## Figures and Tables

**Table 1:** Cancer patients exposed to intravenous bisphosphonates and non-exposed cancer patients matched by cancer type, presence of distant metastasis at diagnosis, age, and gender and the risk of subsequent atrial fibrillation/flutter, Denmark 2000–2008

	**Cancer patients exposed to intravenous bisphosphonates**	**Matched cancer patients with no intravenous bisphosphonate exposure**
Number	3981	7906
Females, *N* (%)	2416 (61)	4804 (61)
Males, *N* (%)	1565 (39)	3102 (39)
Median age at cancer diagnosis (years)	65.5	65.5
		
*Cancer types,* N *(%)*		
Breast cancer	1543 (39)	3083 (39)
Prostate cancer	597 (15)	1194 (15)
Lung cancer	231 (5.8)	462 (5.8)
Kidney cancers	33 (0.8)	65 (0.8)
Multiple myeloma	1021 (26)	2008 (25)
Other cancers	556 (14)	1094 (14)
		
*Metastasis at cancer diagnosis,* N *(%)*		
Distant metastasis	916 (23)	1811 (23)
No distant metastasis	1633 (41)	3256 (41)
Unknown	1432 (36)	2839 (36)
		
*Comorbidities,* N *(%)*		
Cardiovascular disease	826 (21)	1725 (22)
Diabetes	224 (5.6)	426 (5.4)
Pulmonary disease	248 (6.2)	571 (7.2)
Hyperthyroidism	89 (2.2)	181 (2.3)
Alcoholism	92 (2.3)	162 (2.1)
Renal failure	140 (3.5)	470 (5.9)
		
*Treatments in the year before index,* N (%)		
Surgery (any kind)	2841 (71)	4031 (51)
Chemotherapy	2201 (55)	1709 (22)
Radiation therapy	1052 (26)	952 (12)
		
*Atrial fibrillation/flutter*		
Number (%)	128 (3.2)	192 (2.4)
Crude hazard ratio	1.8 (95% CI: 1.4, 2.4)	1.0 (Reference)
Adjusted hazard ratio^a^	1.7 (95% CI: 1.2, 2.4)	1.0 (Reference)

Abbreviation: CI, Confidence interval.

a Adjusted for the comorbidities and treatments listed in the table.

## References

[bib1] Abrahamsen B, Eiken P, Brixen K (2009) Atrial fibrillation in fracture patients treated with oral bisphosphonates. J Intern Med 265(5): 581–592 19141097 10.1111/j.1365-2796.2008.02065.x

[bib2] Andersen TF, Madsen M, Jorgensen J, Mellemkjoer L, Olsen JH (1999) The Danish National Hospital Register. A valuable source of data for modern health sciences. Dan Med Bull 46(3): 263–268 10421985

[bib3] Arslan C, Aksoy S, Dizdar O, Dede DS, Harputluoglu H, Altundag K (2011) Zoledronic acid and atrial fibrillation in cancer patients. Support Care Cancer 19(3): 425–430 20358384 10.1007/s00520-010-0868-z

[bib4] Bhuriya R, Singh M, Molnar J, Arora R, Khosla S (2010) Bisphosphonate use in women and the risk of atrial fibrillation: a systematic review and meta-analysis. Int J Cardiol 142(3): 213–217 20051297 10.1016/j.ijcard.2009.11.041

[bib5] Black DM, Delmas PD, Eastell R, Reid IR, Boonen S, Cauley JA, Cosman F, Lakatos P, Leung PC, Man Z, Mautalen C, Mesenbrink P, Hu H, Caminis J, Tong K, Rosario-Jansen T, Krasnow J, Hue TF, Sellmeyer D, Eriksen EF, Cummings SR (2007) Once-yearly zoledronic acid for treatment of postmenopausal osteoporosis. N Engl J Med 356(18): 1809–1822 17476007 10.1056/NEJMoa067312

[bib6] Bunch TJ, Anderson JL, May HT, Muhlestein JB, Horne BD, Crandall BG, Weiss JP, Lappe DL, Osborn JS, Day JD (2009) Relation of bisphosphonate therapies and risk of developing atrial fibrillation. Am J Cardiol 103(6): 824–828 19268739 10.1016/j.amjcard.2008.11.037

[bib7] Christiansen CF, Christensen S, Mehnert F, Cummings SR, Chapurlat RD, Sorensen HT (2009) Glucocorticoid use and risk of atrial fibrillation or flutter: a population-based, case-control study. Arch Intern Med 169(18): 1677–1683 19822824 10.1001/archinternmed.2009.297

[bib8] Coleman RE, Guise TA, Lipton A, Roodman GD, Berenson JR, Body JJ, Boyce BF, Calvi LM, Hadji P, McCloskey EV, Saad F, Smith MR, Suva LJ, Taichman RS, Vessella RL, Weilbaecher KN (2008) Advancing treatment for metastatic bone cancer: consensus recommendations from the Second Cambridge Conference. Clin Cancer Res 14(20): 6387–6395 18927277 10.1158/1078-0432.CCR-08-1572 PMC2763638

[bib9] Ehrenstein V, Antonsen S, Pedersen L (2010) Existing data sources for clinical epidemiology: Aarhus University prescription database. Clin Epidemiol 2: 273–279 21152254 10.2147/CLEP.S13458 PMC2998817

[bib10] FDA. Update of safety review follow-up to the October 1, 2007 early communication about the ongoing safety review of bisphosphonates. 12 Nov 2008 (online), 3 March 2010, 10 March 2011. http://www.fda.gov/Drugs/DrugSafety/PostmarketDrugSafetyInformationforPatientsandProviders/DrugSafetyInformationforHeathcareProfessionals/ucm136201.htm

[bib11] Frost L, Vestergaard P (2004) Alcohol and risk of atrial fibrillation or flutter: a cohort study. Arch Intern Med 164(18): 1993–1998 15477433 10.1001/archinte.164.18.1993

[bib12] Grosso A, Douglas I, Hingorani A, MacAllister R, Smeeth L (2009) Oral bisphosphonates and risk of atrial fibrillation and flutter in women: a self-controlled case-series safety analysis. PLoS One 4(3): e4720 19266096 10.1371/journal.pone.0004720 PMC2648983

[bib13] Heckbert SR, Li G, Cummings SR, Smith NL, Psaty BM (2008) Use of alendronate and risk of incident atrial fibrillation in women. Arch Intern Med 168(8): 826–831 18443257 10.1001/archinte.168.8.826

[bib14] Howard PA, Barnes BJ, Vacek JL, Chen W, Lai SM (2010) Impact of bisphosphonates on the risk of atrial fibrillation. Am J Cardiovasc Drugs 10(6): 359–367 21090829 10.2165/11584720-000000000-00000

[bib15] Huang WF, Tsai YW, Wen YW, Hsiao FY, Kuo KN, Tsai CR (2010) Osteoporosis treatment and atrial fibrillation: alendronate versus raloxifene. Menopause 17(1): 57–63 19680161 10.1097/gme.0b013e3181b34749

[bib16] Karam R, Camm J, McClung M (2007) Yearly zoledronic acid in postmenopausal osteoporosis. N Engl J Med 357(7): 712–713 17703529

[bib17] Kim SY, Kim MJ, Cadarette SM, Solomon DH (2010) Bisphosphonates and risk of atrial fibrillation: a meta-analysis. Arthritis Res Ther 12(1): R30 20170505 10.1186/ar2938 PMC2875664

[bib18] Loke YK, Jeevanantham V, Singh S (2009) Bisphosphonates and atrial fibrillation: systematic review and meta-analysis. Drug Saf 32(3): 219–228 19338379 10.2165/00002018-200932030-00004

[bib19] Lyles KW, Colon-Emeric CS, Magaziner JS, Adachi JD, Pieper CF, Mautalen C, Hyldstrup L, Recknor C, Nordsletten L, Moore KA, Lavecchia C, Zhang J, Mesenbrink P, Hodgson PK, Abrams K, Orloff JJ, Horowitz Z, Eriksen EF, Boonen S (2007) Zoledronic acid and clinical fractures and mortality after hip fracture. N Engl J Med 357(18): 1799–1809 17878149 10.1056/NEJMoa074941 PMC2324066

[bib20] Mak A, Cheung MW, Ho RC, Cheak AA, Lau CS (2009) Bisphosphonates and atrial fibrillation: Bayesian meta-analyses of randomized controlled trials and observational studies. BMC Musculoskelet Disord 10: 113 19772579 10.1186/1471-2474-10-113 PMC2758833

[bib21] Pedersen CB, Gotzsche H, Moller JO, Mortensen PB (2006) The Danish Civil Registration System. A cohort of eight million persons. Dan Med Bull 53(4): 441–449 17150149

[bib22] Sorensen HT, Christensen S, Mehnert F, Pedersen L, Chapurlat RD, Cummings SR, Baron JA (2008) Use of bisphosphonates among women and risk of atrial fibrillation and flutter: population based case-control study. BMJ 336(7648): 813–816 18334527 10.1136/bmj.39507.551644.BE PMC2292333

[bib23] Sorensen HT, Lash TL, Rothman KJ (2006) Beyond randomized controlled trials: a critical comparison of trials with nonrandomized studies. Hepatology 44(5): 1075–1082 17058242 10.1002/hep.21404

[bib24] Storm HH, Michelsen EV, Clemmensen IH, Pihl J (1997) The Danish Cancer Registry—history, content, quality and use. Dan Med Bull 44(5): 535–539 9408738

[bib25] Vestergaard P, Schwartz K, Pinholt EM, Rejnmark L, Mosekilde L (2010) Risk of atrial fibrillation associated with use of bisphosphonates and other drugs against osteoporosis: a cohort study. Calcif Tissue Int 86(5): 335–342 20309678 10.1007/s00223-010-9349-0

[bib26] Wilkinson GS, Baillargeon J, Kuo YF, Freeman JL, Goodwin JS (2010) Atrial fibrillation and stroke associated with intravenous bisphosphonate therapy in older patients with cancer. J Clin Oncol 28(33): 4898–4905 20940190 10.1200/JCO.2010.28.7524 PMC3020695

[bib27] Woolf AD, Akesson K (2003) Preventing fractures in elderly people. BMJ 327(7406): 89–95 12855529 10.1136/bmj.327.7406.89 PMC1126451

